# Influence of changes in ventricular systolic function and loading conditions on pulse contour analysis-derived femoral d*P*/d*t*_max_

**DOI:** 10.1186/s13613-019-0537-4

**Published:** 2019-05-30

**Authors:** Sergi Vaquer, Denis Chemla, Jean-Louis Teboul, Umar Ahmad, Flora Cipriani, Joan Carles Oliva, Ana Ochagavia, Antonio Artigas, Francisco Baigorri, Xavier Monnet

**Affiliations:** 1 Servei de Medicina Intensiva, Centre de Crítics, Corporació Sanitària Universitària Parc Taulí, Parc Taulí 1, 08208 Sabadell, Spain; 2 Departament de Medicina, Facultat de Medicina, Universitat Autònoma de Barcelona, Passeig de la Vall d´Hebron 119, 08035 Barcelona, Spain; 3 Service de réanimation-médecine intensive, Hôpital Bicêtre, AP-HP, Hôpitaux universitaires Paris-Sud, 78, rue du Général Leclerc, 94270 Le Kremlin-Bicêtre, France; 4 INSERM-UMR_S999 LabEx – LERMIT, Hôpital Marie-Lannelongue, 92350 Le Plessis-Robinson, France; 5 Service d’explorations fonctionnelles multidisciplinaires bi-site Béclère-Bicêtre, AP-HP, Hôpitaux universitaires Paris-Sud, 78, rue du Général Leclerc, 94270 Le Kremlin-Bicêtre, France; 6 Fundació Parc Taulí, Parc Taulí University Institute, Parc Taulí 1, 08208 Sabadell, Spain; 7 Corporacion Sanitaria Universitaria Parc Tauli, CIBER de Enfermedades Respitatorias, Instituto Carlos III, Universitat Autònoma de Barcelona, Parc Taulí 1, 08208 Sabadell, Spain

**Keywords:** Haemodynamic monitoring, Waveform analysis, Ejection fraction, Preload, Afterload, Thermodilution

## Abstract

**Background:**

Femoral d*P*/d*t*_max_ (maximum rate of the arterial pressure increase during systole) measured by pulse contour analysis has been proposed as a surrogate of left ventricular (LV) d*P*/d*t*_max_ and as an estimator of LV systolic function. However, femoral d*P*/d*t*_max_ may be influenced by LV loading conditions. In this study, we evaluated the impact of variations of LV systolic function, preload and afterload on femoral d*P*/d*t*_max_ in critically ill patients with cardiovascular failure to ascertain its reliability as a marker of LV systolic function.

**Results:**

We performed a prospective observational study to evaluate changes in femoral d*P*/d*t*_max_, thermodilution-derived variables (PiCCO2—Pulsion Medical Systems, Feldkirchen, Germany) and LV ejection fraction (LVEF) measured by transthoracic echocardiography during variations in dobutamine and norepinephrine doses and during volume expansion (VE) and passive leg raising (PLR). Correlations with arterial pulse and systolic pressure, effective arterial elastance, total arterial compliance and LVEF were also evaluated. In absolute values, femoral d*P*/d*t*_max_ deviated from baseline by 21% (201 ± 297 mmHg/s; *p* = 0.013) following variations in dobutamine dose (*n* = 17) and by 15% (177 ± 135 mmHg/s; *p* < 0.001) following norepinephrine dose changes (*n* = 29). Femoral d*P*/d*t*_max_ remained unchanged after VE and PLR (*n* = 24). Changes in femoral d*P*/d*t*_max_ were strongly correlated with changes in pulse pressure and systolic arterial pressure during dobutamine dose changes (*R* = 0.942 and 0.897, respectively), norepinephrine changes (*R* = 0.977 and 0.941, respectively) and VE or PLR (*R* = 0.924 and 0.897, respectively) (*p* < 0.05 in all cases). Changes in femoral d*P*/d*t*_max_ were correlated with changes in LVEF (*R* = 0.527) during dobutamine dose variations but also with effective arterial elastance and total arterial compliance in the norepinephrine group (*R* = 0.638 and *R* = − 0.689) (*p* < 0.05 in all cases).

**Conclusions:**

Pulse contour analysis-derived femoral d*P*/d*t*_max_ was not only influenced by LV systolic function but also and prominently by LV afterload and arterial waveform characteristics in patients with acute cardiovascular failure. These results suggest that femoral d*P*/d*t*_max_ calculated by pulse contour analysis is an unreliable estimate of LV systolic function during changes in LV afterload and arterial load by norepinephrine and directly linked to arterial waveform determinants.

**Electronic supplementary material:**

The online version of this article (10.1186/s13613-019-0537-4) contains supplementary material, which is available to authorized users.

## Background

Current haemodynamic monitoring devices performing arterial pulse contour analysis can measure and monitor the maximum rate of rise of arterial pressure (d*P*/d*t*_max_). By analogy with the left ventricle (LV) d*P*/d*t*_max_, arterial d*P*/d*t*_max_ is supposed to reflect LV systolic function [1–8].

Several studies have suggested that measurements of arterial d*P*/d*t*_max_ obtained from radial [7, 9] and femoral [7, 10] arterial pressure waveforms were comparable to LV d*P*/d*t*_max_ and, in some cases, might be useful for predicting patient outcome [11]. However, conflicting results regarding the comparability of LV d*P*/d*t*_max_ and arterial d*P*/d*t*_max_ have also been presented in both adults and children [12, 13]. Despite these uncertainties, arterial d*P*/d*t*_max_ is most often presented as a marker of LV systolic function in many off-the-shelf haemodynamic monitoring systems. Supportive literature is based on the observed good correlation between LV and arterial d*P*/d*t*_max_ during periods of haemodynamic stability [7–9, 12]. However, these good correlations documented on stable patients do not necessarily imply that femoral d*P*/d*t*_max_ provides an adequate evaluation of changes in LV systolic function during haemodynamic challenges.

Many physiological factors other than LV systolic function may influence arterial d*P*/d*t*_max_, including the timing of the measurement relative to aortic valve opening, and the potential influences of cardiac preload and afterload (including its resistive and pulsatile components). To be considered a reliable marker of LV systolic function, arterial d*P*/d*t*_max_ should be unaffected by changes in these variables and should consistently respond to directional changes in LV systolic function.

Therefore, to assess the validity of arterial d*P*/d*t*_max_ as an index of LV systolic function and the relative contribution of changes in cardiac preload and afterload on its measurement, we studied the responses of femoral d*P*/d*t*_max_ during changes in the dose of dobutamine and norepinephrine, during passive leg raising (PLR) manoeuvre [14] and after intravascular fluid administration in critically ill patients with circulatory shock. We also compared these changes with markers of left ventricular afterload and with left ventricle ejection fraction (LVEF) measured by transthoracic echocardiography.

## Methods

We performed a prospective observational study in two adult intensive care units (Servei de Medicina Intensiva, Corporació Sanitària Universitària Parc Taulí, Sabadell, Spain, and Service de Médecine intensive-réanimation, Hôpital de Bicêtre, Le Kremlin-Bicêtre, France). The study was approved by local ethics committees of both institutions (Comitè Ètic d’Investigació Clínica de la Corporació Sanitària Parc Taulí CEIC2013616 and Comité pour la Protection des Personnes Ile-de-France VII 2011A01696-35). All patients or next of kin gave their consent to participate to the study. Data in this manuscript are presented following the “Strengthening the Reporting of Observational Studies in Epidemiology” (STROBE) criteria for observational studies [15].

### Patients

Inclusion criteria were age older than 18 years old and presence of at least one of the following signs of haemodynamic failure in the context of acute illness:

- Systolic arterial pressure ≤ 90 mmHg or decreases of more than 50 mmHg in the last 3 h or mean arterial pressure ≤ 65 mmHg
- Oliguria ≤ 0.5 mL/kg/h for more than 2 h
- Blood lactate ≥ 2 mmol/L (or 22 mg/dL)
- Central venous oxygen saturation ≤ 60%
- Skin mottling

Patients had to be monitored with a transpulmonary thermodilution device (PICCO2, Pulsion Medical Systems, Feldkirchen, Germany) and must present the need for a change in the dose of norepinephrine or dobutamine, or for volume expansion or a PLR test [14], as decided by the attending physicians.

Exclusion criteria were the evidence of a significant aortic stenosis with echocardiography (mean pressure gradient of the aortic valve ≥ 25 mmHg) and conditions precluding measurements of femoral d*P*/d*t*_max_ of sufficient quality such as over-damping or under-damping of the arterial pressure signal persisting after repeated flushes of the arterial line.

### Recorded variables

Arterial pressure was measured through an arterial catheter inserted in the femoral artery (PV2015L20-A, Pulsion Medical Systems, Feldkirchen, Germany). The catheter was connected to a PiCCO2 device, which automatically and continuously calculated femoral d*P*/d*t*_max_. With this device, d*P*/d*t*_max_ was obtained from the uprising portion of the arterial curve, representing the steepest incline of the arterial trace in systole, and averaged over 12 s. After zeroing the arterial pressure transducer system and before each measurement, the arterial waveform signal quality was checked visually using a fast flush test to assess the adequacy of its damping [16]. In case of damping, repeated flushes were performed until sufficient signal quality was acquired. Data were recorded automatically by the PICCO2 device, and synchronisation of measurements with interventions was performed manually and required the presence of the investigator team.

Transthoracic echocardiography was performed with a CX 50 device (Philips Healthcare, DA Best, The Netherlands) and used to estimate LV ejection fraction (LVEF). Measurements were taken by the same observer in all cases (SV) using the Simpson’s method from two- and four-chamber apical views. Endocardial contours were hand-drawn, and volumes were automatically averaged out over three consecutive cardiac cycles by the software to calculate LVEF.

All patients were equipped with a central venous catheter in the superior vena cava territory. Thermodilution measurements were taken by injection of a 15-mL cold saline bolus (< 8 °C) through the central venous catheter. The results of three consecutive thermodilution measurements were averaged [17]. Cardiac output and stroke volume were measured through transpulmonary thermodilution [18] and indexed to body surface to provide cardiac index (CI), stroke volume index (SVI) and global end-diastolic volume index (GEDVi). Cardiac function index was obtained directly from the PiCCO2 device as a calculated variable (CFI = CI/GEDVi) [19].

To evaluate the resistive component of the arterial load, we calculated the systemic vascular resistance index (SVRi) as SVRi = (mean arterial pressure − central venous pressure)/cardiac index. To evaluate the pulsatile component of arterial loading, we calculated the total arterial compliance (TAC = stroke volume/arterial pulse pressure) [20]. Pulse pressure was calculated as the systolic minus the diastolic arterial pressure. The effective arterial elastance was used as a global index of arterial load as previously described (Ea = 0.9 × systolic arterial pressure/stroke volume) [21].

### Study design

Data were collected before and after haemodynamic interventions. Volume expansion was performed by infusing 500 mL 0.9% saline solution over 10 min. Although other fluids might be considered for volume expansion [22], 0.9% saline solution was used in the units at the time the study was performed. A PLR test was performed by moving the patient from the semi-recumbent position to a position where the trunk is horizontal and the legs are elevated at 45°, as previously described [14].

In patients receiving fluid, the post-intervention measurements were taken immediately after the end of volume expansion. In patients in whom a PLR test was performed, these measurements were taken at the time when the maximal PLR-induced change in CI, if any, had occurred. This usually occurs within 1 min [14]. After the change in dose of norepinephrine or dobutamine, the post-interventions recording was performed after stabilisation of pulse contour-derived CI (for dobutamine) or of mean arterial pressure (for norepinephrine).

Patients could be included in the study as many times as therapeutic interventions were indicated by the attending physicians. Multiple measurements on the same patient could only be performed after sufficient time had passed between different manoeuvres to allow for stabilisation of haemodynamic variables and provided that the haemodynamic status of the patient had significantly changed when assessing the same type of interventions.

### Data analysis

During norepinephrine dose variations, changes in Ea, TAC and SVRi were used to identify changes in arterial loading properties, while changes in systolic and mean arterial pressure were used to estimate changes in LV afterload. During dobutamine dose variations, changes in LVEF, CI and CFI were used to estimate changes in LV systolic function. Finally, during PLR and volume expansion, changes in central venous pressure (CVP) and GEDVi were used to track changes in LV preload.

We considered changes in femoral d*P*/d*t*_max_ induced by dobutamine dose variations as the main study variable. Using previous published data [10] and assuming a minimum required threshold of 10%, an *α* risk of 5% and a *β* risk of 20%, we estimated that the minimum number of paired measurements required for detecting a significant change in femoral d*P*/d*t*_max_ during variations in the dose of dobutamine was seven. We continued inclusions in the other study groups (changes in the dose of norepinephrine and PLR/VE) until this number was reached in both dobutamine subgroups (dose increases and decreases).

Normality of variables was assessed using the Kolmogorov–Smirnov test. Data are presented as mean ± standard deviation (SD) or medians and 25th–75th percentile, as appropriate. Data from norepinephrine and dobutamine dose changes were pooled (absolute values of increases and decreases were evaluated together and averaged), and absolute deviations from baseline values (called “changes” or “variations”) were presented as mean differences. Statistical comparisons were made using the paired Student’s *t* test or Wilcoxon rank test as appropriate. Percentages of change, rather than raw values alone, were presented in order to normalise baseline values. Correlation of changes in study variables during interventions was performed using Pearson’s correlation test. In order to evaluate the potential impact of repeated measurements of the same type on a single patient, we studied changes in femoral dP/dt_max_ during interventions using only one measurement per patient. Manoeuvres with the highest norepinephrine or dobutamine dose change were selected, as well as the first volume expansion or PLR performed in each patient. All statistical calculations were done using SPSS version 22 (International Business Machines, Armonk, NY, USA). Values of *p* < 0.05 were considered statistically significant.

## Results

### Patients

Nineteen patients were included (68% male subjects) between March 2013 and January 2015, in whom 72 therapeutic interventions were analysed (162 data points). Arterial line damping problems were observed in five patients, representing nine interventions. In all cases, repeated flushing of the arterial line led to resolution of the damping effect, so that no patient was excluded due to this problem. Two interventions had to be rejected given repeatedly doubtful validity of the data due to patient movement and incorrect acquisition procedure (Additional file 1: Figure S1). The distribution of medical interventions was as follows: norepinephrine dose increase: 9 (13%), norepinephrine dose decrease: 20 (29%), PLR: 12 (17%), volume expansion: 12 (17%), dobutamine dose increase: 7 (10%), dobutamine dose decrease: 10 (14%). On average, 3.7 ± 2.0 interventions were collected in each patient (Additional file 1: Figure S1). Case demographics and clinical characteristics are presented in Table 1 and Additional file 1: Table S1. Baseline haemodynamic characteristics are presented in Table 2. The majority of interventions occurred during septic shock (54 cases; 77%), followed by cardiogenic shock (10 cases; 14%) and hypovolemic shock (6 cases; 9%). During 55 (79%) therapeutic interventions, patients were mechanically ventilated, in 25 (45%) of which patients were not fully adapted to mechanical ventilation. In 44 cases (67%), sinus rhythm was present.

**Table 1:** Baseline demographic and clinical characteristics of included patients

Clinical variable	All patients (*n* = 19)
Weight (kg)	81 ± 19
Height (cm)	166 ± 10
Age (years)	71 ± 9
Apache II (points)	25 ± 10
VT (mL)	406 ± 71
RR (min^−1^)	20 (18–25)
FiO_2_	0.37 ± 0.08
PaO_2_/FiO_2_	257 ± 101
PEEP (cmH_2_O)	6 (5–8)
P_plat_ (cmH_2_O)	19 ± 5
NE (µg kg^−1^ min^−1^)	0.92 ± 0.93
DBT (µg kg^−1^ min^−1^)	5.39 ± 4.9)
WBC (× 10^3^ dL^−1^)	18.6 (11–23)
CRP (mg dL^−1^)	24 ± 11.5
Cr (mg dL^−1^)	2.4 ± 1.2
Bil (mg dL^−1^)	1.1 (0.4–3.4)
Lactate (mg dL^−1^)	48.3 ± 30

Data are presented as mean ± SD or median (25th–75th%)

VT, tidal volume; RR, respiratory rate; FiO_2_, inspired oxygen fraction; PaO_2_, arterial oxygen partial pressure; PEEP, positive end-expiratory pressure; Pplat, plateau pressure; NE, norepinephrine; DBT, dobutamine; WBC, white blood cells; CRP, C reactive protein; Cr, creatinine; Bil, total bilirubin

**Table 2:** Haemodynamic variables at baseline

Haemodynamic variable	DBT (*n* = 17)^a^	NE (*n* = 29)^a^	VE/PLR (*n* = 24)^a^	All interventions (*n* = 70)^a^
Femoral d*P*/d*t*_max_ (mmHg s^−1^)	1049 ± 347	1319 ± 371	1162 ± 336	1199 ± 365
HR (beats min^−1^)	89 ± 14	92 ± 18	90 ± 15	90 (74–104)
SAP (mmHg)	117 ± 17	137 ± 25	123 ± 14	127 ± 21
MAP (mmHg)	75 ± 7	84 ± 16	78 (75–84)	78 (70–86)
PP (mmHg)	66 ± 17	80 ± 18	70 ± 11	73 ± 17
CVP (mmHg)	9 ± 4	9 (8–12)	11 ± 4	9 (8-12)
GEDVi (mL m^−2^)	749 ± 120	773 (684–878)	773 ± 146	750 (671–846)
CI (L min^−1^ m^−2^)	2.7 ± 0.6	3.1 ± 1.2	3 (2.5–3.5)	3 (2.2–3.4)
SVI (mL m^−2^)	31 ± 9	35 ± 14	32 (24–45)	32 (25–44)
CFI (min^−1^)	3.9 ± 1.2	4.3 ± 1.6	4.4 ± 1.7	4.2 ± 1.6
LVEF (%)	43 ± 11	57 (42–61)	54 ± 17	50 ± 14
Ea (mmHg ml^−1^)	2 ± 0.5	2 (1.3–2.4)	1.9 (1.4–2.2)	1.9 (1.4–2.3)
TAC (ml mmHg^−1^)	0.9 ± 0.3	0.8 (0.6–1.2)	0.8 (0.8–1.1)	0.8 (0.7–1.1)
SVRi (dynes s cm^−5^ m^−2^)	1774 (1657–2379)	2241 ± 1078	1739 (1575–2172)^b^	1775 (1627–2404)^b^

Data are presented as mean ± SD or median (25th–75th%)

*NE* norepinephrine, *VE/PLR* volume expansion/passive leg raising, *DBT* dobutamine, *HR* heart rate, *SAP* systolic arterial pressure, *MAP* mean arterial pressure, *PP* pulse pressure, *CVP* central venous pressure, *GEDVi* global end-diastolic volume index, *CI* cardiac index, *SVI* stroke volume index, *CFI* cardiac function index, *LVEF* left ventricle ejection fraction, *Ea* effective arterial elastance, *TAC* total arterial compliance, *SVRi* systemic vascular resistance index

^a^*n* value refers to cases

### Effects of dobutamine

Changes in the dose of dobutamine (*n* = 17 interventions; absolute dose variation = 4.3 ± 1.3 µg kg^−1^ min^−1^) induced an absolute deviation from baseline in femoral d*P*/d*t*_max_ of 21% and were correlated with changes in femoral d*P*/d*t*_max_ (*R* = 0.62; *p* = 0.008). Changes from baseline were also observed in CFI (7%), LVEF (20%), CI (12%) and heart rate (5%) (Table 3). SVI remained unchanged. While systolic arterial pressure and mean arterial pressure values did not vary, pulse pressure changed by 20%. SVRi changed by 5%; however, Ea and TAC presented no significant change. GEDVi and CVP also remained unchanged (Table 3).

**Table 3:** Changes in haemodynamic variables during monitored interventions

Haemodynamic variable	DBT changes		NE changes		VE/PLR	
	Mean difference ± SD	*P* ^a^	Mean difference ± SD	*P* ^a^	Mean difference ± SD	*P* ^a^
Femoral d*P*/d*t*_max_ (mmHg s^−1^)	201 ± 298	*0.013*	177 ± 136	*< 0.001*	59 ± 304	0.355
CFI (min^−1^)	0.3 ± 0.4	*0.013*	0.1 ± 0.2	0.124^b^	0.2 ± 0.4	*0.042*
LVEF (%)	7 ± 5	*< 0.001*	5 ± 13	*0.025* ^b^	− 1 ± 4	0.309^b^
CI (L min^−1^ m^−2^)	0.3 ± 0.5	*0.031*	0.1 ± 0.2	0.112	0.1 ± 0.4	0.153^b^
SVI (mL m^−2^)	0.7 ± 3.7	0.163^b^	1.2 ± 3	*0.040*	1.9 ± 4.1	*0.028* ^b^
HR (beats min^−1^)	4 ± 8	*0.027*	1 ± 3	0.079	− 2 ± 5	*0.019* ^b^
SAP (mmHg)	7 ± 15	0.065	18 ± 14	*< 0.001*	11 ± 22	*0.027*
MAP (mmHg)	3 ± 7	0.089	9 ± 7	*< 0.001*	5 ± 14	0.092^b^
PP (mmHg)	13 ± 10	*< 0.001*	6 ± 12	*< 0.001* ^b^	6 ± 16	0.072
CVP (mmHg)	0 ± 2	0.748	1 ± 2	0.099^b^	3 ± 3	*0.001*
GEDVi (mL m^−2^)	61 ± 244	0.535^b^	3 ± 86	0.380^b^	− 17 ± 74	0.268
Ea (mmHg mL^−1^)	0.05 ± 0.2	0.403	0.18 ± 0.2	*< 0.001* ^b^	0 ± 0.4	0.339^b^
TAC (mL mmHg^−1^)	0.01 ± 0.1	0.696	0.15 ± 0.1	*0.001*	0.03 ± 0.2	0.394^b^
SVRi (dynes s cm^−5^ m^−2^)	94 ± 157	*0.025*	109 ± 210	*0.009*	32 ± 272	0.574

In norepinephrine and dobutamine cases, absolute mean differences are presented. These were calculated as absolute results from increases and decreases in catecholamine dose

*NE* norepinephrine, *VE/PLR* volume expansion/passive leg raising, *DBT* dobutamine, *CFI* cardiac function index, *LVEF* left ventricle ejection fraction, *CI* cardiac index, *SVI* stroke volume index, *HR* heart rate, *SAP* systolic arterial pressure, *MAP* mean arterial pressure, *PP* pulse pressure, *CVP* central venous pressure, *GEDVi* global end-diastolic volume index, *Ea* effective arterial elastance, *TAC* total arterial compliance, *SVRi* systemic vascular resistance index

Significant results (*p* < 0.05) are highlighted in italics

^a^Calculated with Student’s *T* test unless indicated

^b^Calculated with Wilcoxon’s rank test

When only one intervention per patient was considered, changes in the dose of dobutamine induced an absolute deviation from baseline in femoral dP/dt_max_ of 17% (1068 [748–1480] vs. 1254 [812–1672] mmHg^−1^ s^−1^; *n* = 8; *p* = 0.036).

**Fig. 1:**
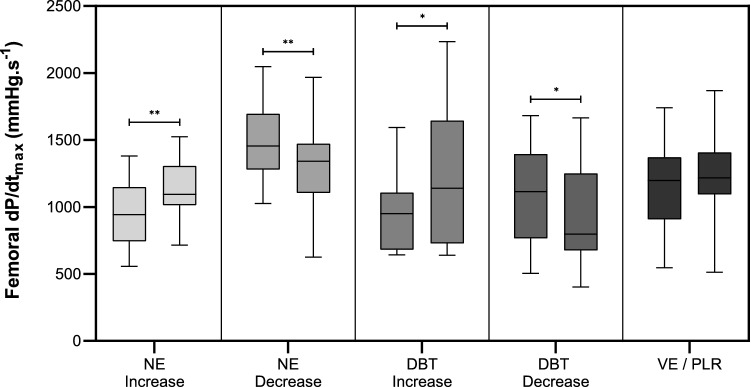
Changes in femoral d*P*/d*t*_max_. Femoral d*P*/d*t*_max_ before and after clinical interventions. Skewers indicate minimum and maximum value. *NE* norepinephrine, *DBT* dobutamine, *VE/PLR* volume expansion, passive leg raising. *p < 0.05; **p < 0.01

Increases in the dose of dobutamine increased femoral d*P*/d*t*_max_ by 20% (Fig. 1; Additional file 1: Table S2) and reductions in the dose led to a decrease in femoral d*P*/d*t*_max_ of 28% (Fig. 1; Additional file 1: Table S2). Additional data from haemodynamic changes obtained before and after increases and decreases in dobutamine doses are presented in Additional file 1: Table S2 in the Supplemental Material.

The dobutamine-induced per cent changes in femoral d*P*/d*t*_max_ were significantly correlated with the per cent changes in CFI, LVEF and CI, but presented the highest correlation with systolic arterial pressure and pulse pressure (Additional file 1: Table S3).

### Effects of changes in norepinephrine dose

Changes in the dose of norepinephrine (*n* = 29 interventions; absolute dose variation = 0.19 ± 0.16 µg kg^−1^ min^−1^) induced an absolute change from baseline in femoral d*P*/d*t*_max_ of 15% and were correlated with changes in femoral d*P*/d*t*_max_ (*R* = 0.47; *p* = 0.011). Arterial systolic, mean and pulse pressure also changed from baseline by 14, 11 and 9%, respectively. There were no significant variations in heart rate, CI and CFI (Table 3). LVEF presented a 11% change from baseline. Although SVI presented a significant change of 4%, CI remained unchanged. CVP and GEDVi also remained at baseline levels. Estimated Ea, TAC and SVRi changed by 9, 17 and 5%, respectively (Table 3).

When only one intervention per patient was considered, changes in the dose of norepinephrine induced an absolute change from baseline in femoral d*P*/d*t*_max_ of 11% (1134 [909–1457] vs. 1265 [1028–1623] mmHg^−1^ s^−1^; *n* = 13; *p* = 0.001).

**Fig. 2:**
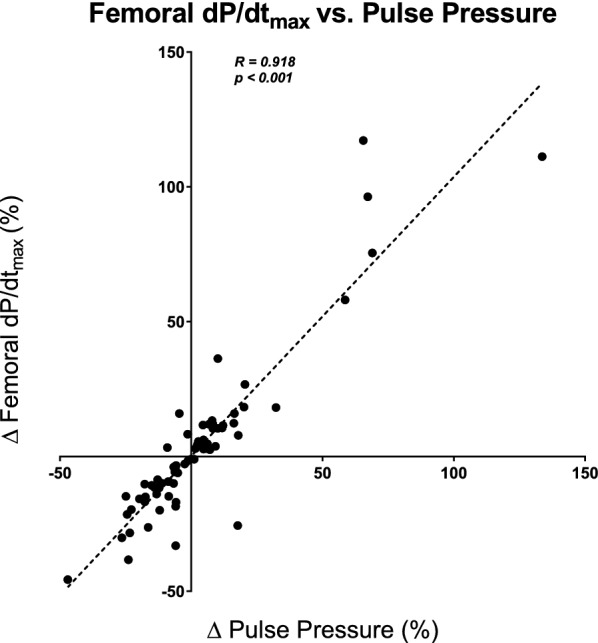
Femoral d*P*/d*t*_max_ versus pulse pressure. Correlation between femoral d*P*/d*t*_max_ and pulse pressure (all interventions pooled)

Increases in the dose of norepinephrine increased femoral d*P*/d*t*_max_ by 16% (Fig. 1 and Additional file 1: Table S2) and reductions in the dose led to a decrease in femoral d*P*/d*t*_max_ of 8% (Fig. 1 and Additional file 1: Table S2). Additional data from haemodynamic changes obtained before and after increases and decreases in norepinephrine doses are presented in Additional file 1: Table S2 (Fig. 2).

The norepinephrine-induced per cent changes in femoral d*P*/d*t*_max_ were correlated with per cent changes in arterial systolic pressure, arterial pulse pressure, Ea and TAC (Additional file 1: Table S3).

### Effects of volume expansion and passive leg raising

The PLR test and volume expansion (*n* = 24 interventions) did not induce significant changes in femoral dP/dt_max_. Heart rate decreased by − 3%, and systolic arterial pressure increased by 9%, while mean arterial pressure and pulse pressure remained unchanged. LVEF did not change, but CFI significantly increased by 4%. In this subgroup, CI did not change from baseline but SVI increased significantly by 5%. CVP increased by 30%, while GEDVi remained at baseline values. Calculated Ea, TAC and SVRi also remained unchanged (Table 3).

When only one intervention per patient was considered, PLR and volume expansion did not modify femoral d*P*/d*t*_max_ (1161 [858–1404] vs. 1218 [1105–1379] mmHg^−1^ s^−1^; *n* = 14; *p* = 0.470).

The PLR and volume expansion-induced changes in femoral d*P*/d*t*_max_ presented the highest correlation with changes in pulse pressure and systolic arterial pressure (Additional file 1: Table S3).

## Discussion

The present study evaluated changes in pulse contour analysis-derived femoral d*P*/d*t*_max_ following haemodynamic interventions aimed at modifying LV systolic function and LV loading conditions (afterload and preload) in critically ill patients with acute circulatory failure. Changes in femoral d*P*/d*t*_max_ were strongly and consistently correlated with changes in arterial pressure waveform determinants across all interventions (arterial systolic pressure and pulse pressure). While femoral d*P*/d*t*_max_ changed during dobutamine infusion, thus suggesting a certain degree of relation with LV systolic function, femoral d*P*/d*t*_max_ also changed during norepinephrine dose variations and was correlated with changes in arterial systolic pressure, pulse pressure, TAC and Ea. Our results suggest that femoral d*P*/d*t*_max_ was mainly sensitive to ventricular loading conditions, specifically afterload, due to arterial load variations, and highly linked to changes in arterial pressure waveform, thus making it an unreliable tool to estimate LV systolic function in acute circulatory failure.

### Femoral d*P*/d*t*_max_ and LV systolic function

Pulse contour analysis is used at the bedside for estimating several haemodynamic variables. In particular, the arterial d*P*/d*t*_max_ is automatically displayed and is thought by many to be an indicator of LV systolic function. As expected, femoral d*P*/d*t*_max_ changed following dobutamine increases and decreases and was related to the direction and magnitude of the dose variation. Furthermore, although LVEF and CFI are not pure estimators of LV systolic function, femoral d*P*/d*t*_max_ changed coherently with these markers during dobutamine dose variations. Note that we assessed the LV systolic function not only with CFI, which is only an estimation of LVEF and which might be mathematically coupled with GEDVi [23], but also more directly, with LVEF measured by echocardiography. These results would suggest that femoral d*P*/d*t*_max_ retains a certain degree of relationship with LV systolic function. Also, it has been previously observed in animal models that LV d*P*/d*t*_max_ reflects cardiac contractility when adequate LV filling is achieved [24, 25]. In our study, femoral d*P*/d*t*_max_ remained unchanged during VE or PLR, which could be explained by an optimised LV preload status at the time of the intervention. This finding would suggest that, similarly to what has been previously observed [24, 25], femoral d*P*/d*t*_max_ is independent from cardiac preload changes, as it would be expected from a marker of LV systolic function.

### Femoral d*P*/d*t*_max_, LV afterload and arterial load

Our results also indicated that femoral d*P*/d*t*_max_ is markedly influenced by changes in LV afterload (as estimated by changes in systolic and mean arterial pressure) during changes in the dose of norepinephrine. Unlike LV d*P*/d*t*_max_, which is measured during the isovolumetric phase of LV contraction before aortic valve opening [2], femoral d*P*/d*t*_max_ takes place during the LV ejection phase and should therefore be more sensitive to changes in LV afterload. Our results support this hypothesis by evidencing not only significant changes in femoral d*P*/d*t*_max_ during changes in norepinephrine dose, but also significant correlations between changes in femoral d*P*/d*t*_max_ and changes in systolic and mean arterial pressure during such interventions. This demonstrates a dependence of femoral d*P*/d*t*_max_ with LV afterload, which may invalidate its utility as a marker of LV systolic function.

An interesting additional finding of the present study was the strong linear correlation observed between determinants of the arterial pressure waveform and femoral d*P*/d*t*_max_. Our results indicate that femoral d*P*/d*t*_max_ maintained an almost one-to-one relationship with arterial pulse pressure and systolic arterial pressure, meaning that any change in the latter inevitably led to a change in the former. In other words, the higher the amplitude of the arterial waveform, the higher the velocity of the pressure increase, provided that heart rate remains almost constant and vice versa (constant cardiac cycle duration). As observed in our results, this relationship was strong and was observed even in cases where arterial loading conditions remained unchanged, such as during volume expansion and PLR. Therefore, any haemodynamic change affecting pulse pressure and systolic arterial pressure should, in principle, affect femoral d*P*/d*t*_max_ without any corresponding changes in LV contractility. It has been previously described that arterial system compliance, pulse wave reflection and arterial system impedance affect the peripheral arterial waveform [26–29]. We did not study pulse reflection waves in our patients, but we were able to confirm this hypothesis in our study by identifying a strong correlation of femoral dP/dt_max_ with determinants of arterial load (as estimated by Ea, TAC and SVRi) during norepinephrine dose variations.

### Femoral d*P*/d*t*_max_ in clinical practice

Our study challenges the previous belief that femoral d*P*/d*t*_max_ could be used as a reliable marker of LV systolic function at the bedside. This belief was based on the observed good correlation between LV and arterial d*P*/d*t*_max_ during periods of haemodynamic stability [7, 9, 12]. However, correlations alone lack the sufficient value to inform on the responses of femoral d*P*/d*t*_max_ to treatments during cardiovascular failure. The evaluation of dynamic changes during haemodynamic challenges in our study demonstrates that although femoral d*P*/d*t*_max_ is not completely independent from changes in LV systolic function, it is significantly affected by peripheral arterial properties and waveform characteristics.

Previous reports have also identified a strong relationship between femoral d*P*/d*t*_max_ and LV d*P*/d*t*_max_ during isolated changes in LV systolic function, independently from changes in LV loading conditions [10, 30]. In a recent study on healthy animals, Monge Garcia et al. [31] presented a thorough evaluation of arterial d*P*/d*t*_max_ and its relation to LV d*P*/d*t*_max_ and other markers of LV systolic function during changes in cardiac inotropic state, preload and afterload. Authors documented a positive relationship between femoral d*P*/d*t*_max_ and changes in LV systolic function, but also reported + 24% and − 33% changes in femoral d*P*/d*t*_max_ during increases or decreases in LV afterload induced by epinephrine and nitroprusside infusion, respectively, and a 20% reduction in femoral d*P*/d*t*_max_ during acute preload reductions induced by bleeding. Although authors conclude that the most relevant factor of femoral d*P*/d*t*_max_ was the change in LV systolic function, these observations also show the relevant effect of loading conditions on femoral d*P*/d*t*_max_ and corroborate our findings.

Therefore, it is only in cases in which one could reasonably expect that arterial loading properties and LV afterload are unchanged, that LV systolic function is the only factor modified and that femoral d*P*/d*t*_max_ might be used as a marker of LV systolic function. It must be admitted that such cases are uncommon in a constantly changing critically ill patient.

## Limitations

The present study has some limitations that warrant further discussion. First, the number of cases was small and the inclusion rate slow due to the need for specific recording equipment and need for manual synchronisation between interventions and data acquisition. Second, we did not compare measurements of femoral d*P*/d*t*_max_ with LV d*P*/d*t*_max_. Nevertheless, the objective of the present study was to evaluate the responses of femoral d*P*/d*t*_max_ during haemodynamic challenges, and values of LV d*P*/d*t*_max_ would not have helped to fulfil such objective. Furthermore, LV catheterisation for the only purpose of the study would not have been acceptable from an ethical point of view. Alternatively, the estimation of LV d*P*/d*t*_max_ by echocardiography could have been performed. However, such an estimation at the bedside in critically ill patients is far from easy and may have provided unreliable measurements. Third, we did not use any device to evaluate and compensate damping of the arterial pressure signal as utilised by previous authors [7]. However, such devices present their highest utility when high resolution of the arterial waveform is required, for example, for resonance wave analyses, which was not the case in our study. Furthermore, the absence of under- and over-damping phenomena was checked at the beginning of recordings. Fourth, while repetition of measurements on the same patient could be considered as a source of bias, we obtained the same pattern of responses to clinical interventions when only one measurement per patient was evaluated. Fifth, in order to obtain better information of potential causality and to homogeneously spread the interventions across patients, it would have been better to have followed a crossover interventional study design. However, this would have been unethical, since patients would have had to receive intravenous fluids, norepinephrine and dobutamine regardless of any clinical indication to receive such treatments. Sixth, respiratory cycle variations may alter LVEF. This potential source of bias was not taken into account when performing measurements. However, LVEF measurements were averaged over three cardiac cycles, which attenuated any respiratory variation. Furthermore, measurements were obtained during periods of haemodynamic stability and under controlled mechanical ventilation or non-distressed spontaneous ventilation, such that the respiratory variation of LVEF was probably negligible. Finally, a potential mathematical coupling between the measurement of femoral d*P*/d*t*_max_ and systolic arterial pressure or pulse pressure could be a point of concern. However, with the PiCCO2 device used in our study, femoral d*P*/d*t*_max_ was calculated at the moment of maximal pressure rise in the systolic phase of the arterial curve and was not averaged during a time segment of the curve. This approach likely discarded any potential mathematical coupling.

## Conclusions

Femoral d*P*/d*t*_max_ calculated by pulse contour analysis is an unreliable estimate of LV systolic function as it is markedly sensitive to LV afterload variations and changes in arterial loading properties during acute changes in norepinephrine, and directly linked to arterial waveform characteristics.

## Additional file

**Additional file 1.** This file contains a patient and interventions flow chart, additional information on population characteristics, pre- and post-intervention values of haemodynamic variables for the norepinephrine and dobutamine groups, as well as a correlation matrix between femoral d*P*/d*t*_max_ and other haemodynamic variables during studied interventions.

## Data Availability

The datasets used and/or analysed during the current study are available from the corresponding author upon request.

## Abbreviations

LV: left ventricle
PLR: passive leg raising
LVEF: left ventricle ejection fraction
CI: cardiac index
SVI: stroke volume index
GEDVi: global end-diastolic volume index
SVRi: systemic vascular resistance index
TAC: total arterial compliance
Ea: effective arterial elastance
CVP: central venous pressure
SD: standard deviation

## References

[1] Teboul J-L, Saugel B, Cecconi M (2016). Less invasive hemodynamic monitoring in critically ill patients. Intensive Care Med.

[2] Quinones M, Gaasch WH, Alexander JK (1976). Influence of acute changes in preload, afterload, contractile state and heart rate on ejection and isovolumic indices of myocardial contractility in man. Circulation.

[3] Bargiggia GS, Bertucci C, Recusani F (1989). A new method for estimating left ventricular d*P*/d*t* by continuous wave Doppler-echocardiography. Validation studies at cardiac catheterization. Circulation.

[4] Chen C, Rodriguez L, Guerrero JL (1991). Noninvasive estimation of the instantaneous first derivative of left ventricular pressure using continuous-wave Doppler echocardiography. Circulation.

[5] Chen C, Rodriguez L, Lethor JP (1994). Continuous wave Doppler echocardiography for noninvasive assessment of left ventricular d*P*/d*t* and relaxation time constant from mitral regurgitant spectra in patients. J Am Coll Cardiol.

[6] Sugawara M, Senda S, Katayama H (1994). Noninvasive estimation of left ventricular Max(d*P*/d*t*) from aortic flow acceleration and pulse wave velocity. Echocardiography.

[7] Scolletta S, Bodson L, Donadello K (2013). Assessment of left ventricular function by pulse wave analysis in critically ill patients. Intensive Care Med.

[8] Yang F, Iacobelli R, Ming J (2018). Assessment of cardiac function in infants with transposition of the great arteries after surgery: comparison of two methods. World J Pediatr.

[9] Tartiere J-M, Logeart D, Beauvais F (2007). Non-invasive radial pulse wave assessment for the evaluation of left ventricular systolic performance in heart failure. Eur J Heart Fail.

[10] De Hert SG, Robert D, Cromheecke S (2006). Evaluation of left ventricular function in anesthetized patients using femoral artery d*P*/d*t*(max). J Cardiothorac Vasc Anesth.

[11] Tartière JM, Tabet JY, Logeart D (2008). Noninvasively determined radial d*P*/d*t* is a predictor of mortality in patients with heart failure. Am Heart J.

[12] Kim J, Bang J, Park CS (2016). Usefulness of the maximum rate of pressure rise in the central and peripheral arteries after weaning from cardiopulmonary bypass in pediatric congenital heart surgery. Medicine (Baltimore).

[13] Sharman JE, Qasem AM, Hanekom L (2007). Radial pressure waveform d*P*/d*t* max is a poor indicator of left ventricular systolic function. Eur J Clin Invest.

[14] Monnet X, Teboul J-L (2015). Passive leg raising: five rules, not a drop of fluid!. Crit Care.

[15] Von Elm E, Altman DG, Egger M (2007). The strengthening the reporting of observational studies in epidemiology (STROBE) statement: guidelines for reporting observational studies. Ann Intern Med.

[16] Hofkens P-J, Verrijcken A, Merveille K (2015). Common pitfalls and tips and tricks to get the most out of your transpulmonary thermodilution device: results of a survey and state-of-the-art review. Anestezjol Intens Ter.

[17] Monnet X, Persichini R, Ktari M (2011). Precision of the transpulmonary thermodilution measurements. Crit Care.

[18] Monnet X, Teboul J-L (2017). Transpulmonary thermodilution: advantages and limits. Crit Care.

[19] Jabot J, Monnet X, Bouchra L (2009). Cardiac function index provided by transpulmonary thermodilution behaves as an indicator of left ventricular systolic function. Crit Care Med.

[20] Chemla D, Hébert JL, Coirault C (1998). Total arterial compliance estimated by stroke volume-to-aortic pulse pressure ratio in humans. Am J Physiol.

[21] Chemla D, Antony I, Lecarpentier Y (2003). Contribution of systemic vascular resistance and total arterial compliance to effective arterial elastance in humans. Am J Physiol.

[22] Malbrain MLNG, Van Regenmortel N, Saugel B (2018). Principles of fluid management and stewardship in septic shock: it is time to consider the four D’s and the four phases of fluid therapy. Ann Intensive Care.

[23] Malbrain MLNG, De Potter TJR, Dits H (2010). Global and right ventricular end-diastolic volumes correlate better with preload after correction for ejection fraction. Acta Anaesthesiol Scand.

[24] Morimont P, Lambermont B, Desaive T (2012). Arterial d*P*/d*t* max accurately reflects left ventricular contractility during shock when adequate vascular filling is achieved. BMC Cardiovasc Disord.

[25] Blaudszun G, Licker MJ, Morel DR (2013). Preload-adjusted left ventricular d*P*/d*t* max: a sensitive, continuous, load-independent contractility index. Exp Physiol.

[26] Klabunde RE (2012). Cardiovascular physiology concepts.

[27] William MDG, Baim DS. Grossman’s cardiac catheterization, angiography, and intervention, 6th ed; 2000.

[28] Lamia B, Chemla D, Richard C (2005). Clinical review: interpretation of arterial pressure wave in shock states. Crit Care.

[29] Hashimoto J, Ito S (2010). Pulse pressure amplification, arterial stiffness, and peripheral wave reflection determine pulsatile flow waveform of the femoral artery. Hypertension.

[30] Masutani S, Iwamoto Y, Ishido H (2009). Relationship of maximum rate of pressure rise between aorta and left ventricle in pediatric patients. Implication for ventricular-vascular interaction with the potential for noninvasive determination of left ventricular contractility. Circ J.

[31] Garcia MI (2018). Performance comparison of ventricular and arterial d*P*/d*t* max for assessing left ventricular systolic function during different experimental loading and contractile conditions. Crit Care.

