# Supplementary material for: Influence of changes in ventricular systolic function and loading conditions on pulse contour analysis-derived femoral dP/dtmax
Source: Ann Intensive Care. 2019 May 30;9:61. doi: 10.1186/s13613-019-0537-4 (PMC6542880; doi:10.1186/s13613-019-0537-4)
Supplement: Supplementary file 1 — Additional file 1. This file contains a patient and interventions flow chart, additional information on population characteristics, pre- and post-intervention values of haemodynamic variables for the norepinephrine and dobutamine groups, as well as a correlation matrix between femoral dP/dtmax and other haemodynamic variables during studied interventions. [file 13613_2019_537_MOESM1_ESM.docx]

**Supplemental Electronic Material**

**Influence of changes in ventricular systolic function and loading conditions on pulse contour analysis-derived femoral dP/dt_max_**

**Authors**

VAQUER Sergi, MD; CHEMLA Denis, PhD; TEBOUL Jean-Louis, PhD; AHMAD Umar, MD; CIPRIANI Flora, MD; OLIVA Joan Carles, OCHAGAVIA Ana, MD; ARTIGAS Antonio, PhD; BAIGORRI Francisco, PhD; MONNET Xavier, PhD.

**Figure S1 – Flow chart of included patients and collected interventions**

NE : norepinephrine, DBT ; dobutamine, VE/PLR : volume expansion, passive leg raising..

**Table S1 - Population characteristics - Diagnosis**

| **Diagnosis** | **n^a^** | **%** |
| --- | --- | --- |
| Pneumonia | 20 | 29 |
| Soft Tissue | 24 | 34 |
| UTI | 4 | 6 |
| Colecystitis | 2 | 3 |
| Peritonitis | 8 | 11 |
| Pancreatitis | 6 | 9 |
| Cardiac Arrest | 6 | 9 |
|  |  |  |
| Total | 70 | 100 |

^a^ n refers to number of cases

UTI: Urinary Track Infection

**Table S2: Before and After values of Haemodynamic Variables**

|  | **DBT up (n = 7)** | | | **DBT down (n = 10)** | | |
| --- | --- | --- | --- | --- | --- | --- |
|  | Pre  median (25-75%) | Post  median (25-75%) | p | Pre  median (25-75%) | Post  median (25-75%) | p |
| Femoral dP/dt_max_  (mmHg.s^-1^) | 950 (681-1107) | 1140 (728-1646) | **0.043** | 1114 (766-1395) | 797 (677-1250) | **0.017** |
| CFI (min^-1^) | 3.2 (2.3-4.5) | 4.1 (2.5-5) | 0.066 | 4.4 (3.5-5.1) | 4 (3-4.9) | 0.058 |
| LVEF (%) | 37 (30-45) | 37 (34-55) | **0.043** | 51 (36-53) | 36 (33-46) | **0.005** |
| CI (L.min^-1^.m^-2^) | 2.6 (1.9-2.9) | 2.9 (2-3.1) | **0.027** | 2.9 (2.4-3.4) | 2.6 (2.3-3.1) | 0.069 |
| SVI (mL.m^-2^) | 26 (23-28) | 28 (23-31) | 0.345 | 29 (27-43) | 31 (26-38) | 0.285 |
| HR (beats.min^-1^) | 94 (79-100) | 96 (80-105) | **0.028** | 88 (73-105) | 77 (72-96) | 0.059 |
| SAP (mmHg) | 108 (97-119) | 116 (99-127) | 0.398 | 123 (102-141) | 111 (101-127) | 0.059 |
| PP (mmHg) | 61 (49-70) | 67 (51-76) | 0.310 | 68 (52-92) | 60 (49-81) | **0.047** |
| Ea (mmHg.ml^-1^) | 2.1 (1.9-2.3) | 2.1 (1.7-2.3) | 0.249 | 1.8 (1.5-2.2) | 2 (1.5-2.4) | 0.959 |
| C (ml.mmHg^-1^) | 0.7 (0.7-1) | 0.8 (0.7-1.1) | 0.753 | 0.9 (0.7-1) | 0.9 (0.8-1.1) | 0.285 |
| SVRI (dynes.s.cm^-5^.m^-2^) | 1729 (1633-2867) | 1697 (1516-2725) | 0.128 | 1782 (1663-2193) | 1925 (1693-2277) | **0.013** |
|  | **NA up (n = 9)** | | | **NA down (n = 20)** | | |
|  | Pre  median (25-75%) | Post  median (25-75%) | p | Pre  median (25-75%) | Post  median (25-75%) | p |
| Femoral dP/dt_max_  (mmHg.s^-1^) | 943 (743-1148) | 1093 (1013-1306) | **0.008** | 1455 (1280-1696) | 1341 (1106-1473) | **< 0.001** |
| CFI (min^-1^) | 4.2 (3.2-5.4) | 4.1 (3.1-5.5) | 0.831 | 3.7 (3.3-5.2) | 3.7 (3.1-5.2) | 0.131 |
| LVEF (%) | 53 (47-63) | 45 (39-61) | 0.197 | 59 (39-61) | 60 (43-62) | 0.124 |
| CI (L.min^-1^.m^-2^) | 3.3 (2.3-4.1) | 3.4 (2.3-4) | 0.767 | 3.1 (1.9-3.6) | 3.1 (1.9-3.4) | 0.093 |
| SVI (mL.m^-2^) | 47 (28-54) | 48 (27-55) | 0.678 | 31 (19-37) | 29 (19-37) | **0.006** |
| HR (beats.min^-1^) | 75 (72-96) | 75 (72-97) | 0.767 | 97 (80-108) | 97 (81-110) | 0.085 |
| SAP (mmHg) | 108 (101-119) | 136 (113-139) | **0.008** | 147 (138-165) | 127 (112-150) | **< 0.001** |
| PP (mmHg) | 57 (54-73) | 83 (63-87) | **0.008** | 87 (80-100) | 73 (64-86) | **< 0.001** |
| Ea (mmHg.ml^-1^) | 1.27 (0.82-2) | 1.27 (1-2.2) | **0.028** | 2.3 (1.6-3.5) | 2 (1.5-3.5) | **0.004** |
| C (ml.mmHg^-1^) | 1.27 (0.8-1.8) | 1.2 (0.7-1.5) | **0.011** | 0.7 (0.4-1) | 0.8 (0.4-1.1) | **< 0.001** |
| SVRI (dynes.s.cm^-5^.m^-2^) | 1450 (958-1998) | 1386 (1184-2111) | 0.260 | 2159 (1714-3669) | 2115 (1743-3573) | **0.002** |

CFI: Cardiac Function Index; LVEF: Left Ventricle Ejection Fraction; CI: Cardiac Index; SVI: Stroke Volume Index; HR: Heart Rate; SAP: Systolic Arterial Pressure; PP: Pulse Pressure; Ea: Effective Arterial Elastance; C: Total Arterial Compliance; SVRi: Systemic Vascular Resistance Index.

Due to limited sample size in certain groups non-parametric Wilcoxon Rank – Test was used to compare variables pre-post intervention.

**Table S3: Correlation matrix of changes in Femoral dP/dt_max_ vs. changes in main haemodynamic variables**

| Intervention | ΔCFI (%) | ΔLVEF (%) | ΔCI (%) | ΔSVI (%) | ΔHR (%) | ΔSAP (%) | ΔMAP (%) | ΔPP (%) | ΔCVP(%) | ΔGEDI (%) | ΔEa (%) | ΔTAC (%) | ΔSVRI (%) |
| --- | --- | --- | --- | --- | --- | --- | --- | --- | --- | --- | --- | --- | --- |
| NE change  (n=29) | 0.147 ^ns^ | -0.685 ^(2)^ | 0.305 ^ns^ | 0.312 ^ns^ | 0.038 ^ns^ | 0.941 | 0.838 | **0.977** | 0.174 ^ns (2)^ | 0.193 ^ns^ | 0.638 | -0.689 | 0.588 |
| VE/PLR  (n=24) | 0.449 | 0.098 ^ns (3)^ | 0.599 | 0.625 | -0.082 ^ns^ | 0.897 | 0.786 | **0.924** | 0.298 ^ns (3)^ | 0.484 | 0.299 ^ns^ | -0.514 | 0.234 ^ns^ |
| DBT change  (n=17) | 0.585 | 0.527 | 0.597 | 0.409 ^ns^ | 0.147 ^ns^ | 0.897 | 0.826 | **0.942** | 0.317 ^ns (1)^ | 0.021 ^ns^ | -0.199 ^ns^ | -0.148 ^ns^ | 0.088 ^ns^ |
| ALL  (n=70) | 0.405 | 0.114 ^ns (5)^ | 0.522 | 0.488 | 0.009 ^ns^ | 0.884 | 0.776 | **0.918** | 0.304 ^(4)^ | 0.260 | 0.360 | -0.543 | 0.332 |

NE: Norepinephrine; VE/PLR: Volume Expansion / Passive Leg Raising; DBT: Dobutamine; CFI: Cardiac Function Index; LVEF: Left Ventricle Ejection Fraction; CI: Cardiac Index; SVI: Stroke Volume Index; HR: Heart Rate; SAP: Systolic Arterial Pressure; MAP: Mean Arterial Pressure; PP: Pulse Pressure; CVP: Central Venous Pressure; GEDI; Global End Diastolic Index; Ea: Effective Arterial Elastance; TAC: Total Arterial Compliance; SVRi: Systemic Vascular Resistance Index.

Values represent the Pearson’s correlation coefficients.

Δ indicates changes from baseline.

All correlations are significant (p < 0.05) unless indicated (ns).

The best correlation coefficient for each intervention is highlighted in Bold.

(1): n = 11; (2): n = 19; (3): n = 17; (4) n = 47; (5): n = 53
